# Clinical Usefulness of Ultrasound-Guided Fine Needle Aspiration and Core Needle Biopsy for Patients with Axillary Lymphadenopathy

**DOI:** 10.3390/medicina57070722

**Published:** 2021-07-16

**Authors:** Tomoyuki Fujioka, Mio Mori, Kazunori Kubota, Emi Yamaga, Yuka Yashima, Goshi Oda, Tsuyoshi Nakagawa, Iichiroh Onishi, Toshiyuki Ishiba, Ukihide Tateishi

**Affiliations:** 1 Department of Diagnostic Radiology, Tokyo Medical and Dental University, 1-5-45 Yushima, Bunkyo-ku, Tokyo 113-8510, Japan; fjokmrad@tmd.ac.jp (T.F.); ymgdrnm@tmd.ac.jp (E.Y.); 11.ruby.89@gmail.com (Y.Y.); ttisdrnm@tmd.ac.jp (U.T.); 2 Department of Radiology, Dokkyo Medical University, 880 Kitakobayashi, Mibu, Shimotsugagun, Tochigi 321-0293, Japan; kubotard@dokkyomed.ac.jp; 3 Department of Surgery, Breast Surgery, Tokyo Medical and Dental University, 1-5-45 Yushima, Bunkyo-ku, Tokyo 113-8510, Japan; oda.srg2@tmd.ac.jp (G.O.); nakagawa.srg2@tmd.ac.jp (T.N.); 4 Department of Diagnostic Pathology, Tokyo Medical and Dental University, 1-5-45 Yushima, Bunkyo-ku, Tokyo 113-8510, Japan; iichpth2@tmd.ac.jp; 5 Department of Breast Surgery, Tokyo Metropolitan Cancer and Infectious Diseases Center Komagome Hospital, 3-18-22 Honkomagome, Bunkyo-ku, Tokyo 113-8677, Japan; ishiba0313@gmail.com

**Keywords:** ultrasound, core needle biopsy, fine needle aspiration, axillary lymph nodes, lymphadenopathy

## Abstract

*Background and Objectives*: It is necessary to properly diagnose and manage axillary lymphadenopathy caused by a variety of diseases. This study aimed to evaluate the utility of ultrasound (US)-guided sampling in patients with axillary lymphadenopathy. *Materials and Methods*: Patients with axillary lymphadenopathy (excluding patients with newly diagnosed breast cancer) who underwent US-guided fine needle aspiration (FNA) or core needle biopsy (CNB) at a single center between February 2016 and September 2020 were retrospectively examined. The association between US imaging findings and malignancy was investigated and the diagnostic performance of US-guided sampling was assessed. *Results*: Fifty-five patients (including eight males) were included in the study; of these, 34 patients (61.8%) were finally diagnosed with a malignant lymph node lesion. Twenty-two patients (40.0%) had undergone FNA and 33 (60.0%) had undergone CNB. Larger short and long axis diameters, thicker lymph node cortex, and the absence of fatty hilum on the US were significantly associated with malignancy (*p* < 0.05). The diagnostic performance of FNA, CNB, and FNA + CNB was excellent (sensitivity, specificity, and accuracy of 0.909, 0.900, and 0.917 for FNA, 0.958, 1.000, and 0.970 for CNB, and 0.941, 0.952, and 0.945 for FNA + CNB, respectively). *Conclusions*: US-guided FNA and CNB play an important role in the diagnosis and management of patients with axillary lymphadenopathy.

## 1. Introduction

Ultrasound (US) is a primary noninvasive diagnostic modality for evaluating axillary lymphadenopathy. US is particularly useful for assessing the status of axillary lymph nodes as part of preoperative staging, therapy evaluation, and post-treatment surveillance of patients with breast cancer [1,2]. Metastasis of breast cancer is the most common cause of axillary lymphadenopathy; however, axillary lymphadenopathy is also caused by metastasis of other malignant tumors, malignant lymphoma, hematologic malignancies, benign reactive nodes, lymphadenitis, sarcoidosis, and connective tissue diseases [3].

Several ultrasonographic criteria are used to characterize malignant lymph nodes, such as size, shape, cortical thickening, and absence of fatty hilum; however, typical US signs of malignant lesions may also be observed in benign entities [4,5].

Clinical information and US findings can help evaluate the malignant potential of lymph node lesions, based on which, US-guided sampling can help medical professionals to arrive at a definitive diagnosis [6,7,8]. Recently, remarkable advances have occurred in chemotherapy and molecular targeted therapy [9]. The appropriate diagnosis of malignant lesions in axillary lymph nodes can help provide effective treatment. Confirmation of the absence of disease in the axillary lymph nodes can also prevent overtreatment.

US-guided sampling is widely used for the histological diagnosis of axillary lymphadenopathy because of its minimally invasive nature and safety [7,8]. Several studies have documented the usefulness of US-guided fine needle aspiration (FNA) and core needle biopsy (CNB) for staging lymph node metastasis of breast cancer [7,8]; however, the efficacy of these procedures for evaluating axillary lymphadenopathy caused by other diseases is not well characterized [6,10,11].

In this study, patients with axillary lymphadenopathy who underwent US-guided sampling were investigated. The association of clinical findings and US imaging findings with malignancy was examined and the diagnostic performance of US-guided FNA and CNB in distinguishing between benign and malignant lymph node lesions was assessed. Furthermore, the role of US-guided FNA and CNB in the diagnosis and management of patients with lymphadenopathy was discussed.

## 2. Materials and Methods

### 2.1. Study Population

This retrospective study was approved by the medical ethics committee of our institution; the requirement for written informed consent of patients was waived. The inclusion criteria were: (a) patients who underwent US examination and were diagnosed with axillary lymphadenopathy; (b) those who underwent US-guided FNA or CNB of the lymph node between February 2016 and September 2020; and (c) those with a history of breast cancer surgery, being disease free for at least 2 years. Patients with newly diagnosed breast cancer were excluded. US examinations performed at other facilities were excluded in order to standardize image quality.

After reviewing the radiology reports database and clinical records at our institute, two radiologists (with 11 years and 4 years of imaging experience) retrieved the US images, clinical information, and histopathological results of patients who underwent US-guided FNA or CNB during the study period. A comprehensive final diagnosis of benign or malignant condition was made based on imaging findings, histopathology results, and subsequent disease course.

### 2.2. US Examinations and US-Guided Sampling

US examinations were performed by one of the five board-certified radiologists with 5–20 years of experience in breast US using Aplio XG scanner with a PLT-805AT 8.0-MHz linear probe (Toshiba Medical Systems, Tochigi, Japan) or Aplio 500 scanner with a PLT-805AT 8.0-MHz linear probe (Toshiba Medical Systems, Tochigi, Japan).

The patients lay supine on the examination bed with their arms raised. Transverse and longitudinal static images were obtained and the maximum diameter of the lymph node was measured. Subsequently, the radiologist evaluated the axillary lymph nodes by B-mode examination and decided whether to perform US-guided sampling. The choice between US-guided FNA or CNB was at the discretion of the radiologist. For patients with more than two enlarged lymph nodes, the lymph node with the higher suspicion index was selected for sampling. FNA was performed using a 23-gage needle without anesthetic. CNB was performed using a Bard Magnum biopsy system (Bard Biopsy, Tempe, Arizona, AZ, USA) with 16-gage needle with a 15- or 22-mm throw following infiltration with 1% xylocaine. FNA and CNB were typically performed once and three times, respectively; however, additional sampling was performed if the sample volume was inadequate.

### 2.3. Pathological Evaluation

FNA samples were immediately fixed in anhydrous ethanol. All FNA smear slides were stained with Papanicolaou stain. FNA samples were evaluated for the presence of malignant cells by more than two pathologists and two cytologists. The diagnostic FNA cytology classification was as follows: class I = benign; class II = probably benign; class III = equivocal; class IV = probably malignant; and class V = malignant. In this study, the definition of cytological malignancy included classes IV and V and benign lesions were defined as classes I and II. CNB specimens were immediately placed in 10% formalin and embedded in paraffin after fixation. The samples were cut into 3-μm thick slices and then stained with hematoxylin-eosin. CNB specimens were evaluated by more than two pathologists. Immunohistochemical staining was performed at the discretion of the pathologists.

### 2.4. Image Analysis

**Figure 1:**
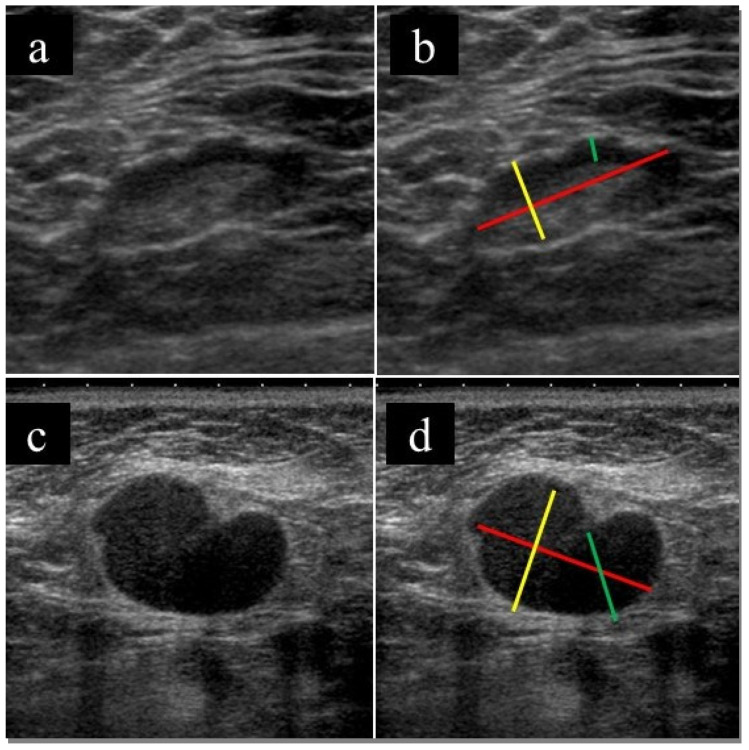
Example of the assessment method for lymph nodes. (**a**,**c**) Original US images and (**b**,**d**) US images after assessment of lymphonodes. The red line shows the major axis diameter, the yellow line shows the minor axis diameter, and the green line shows the thickness of the cortex (**b**,**d**). Fatty hilum is present (**a**), and fatty hilum is absent (**c**).

US imaging findings of the lymph nodes were retrospectively evaluated by a breast radiologist (with 11 years of experience); the evaluator was blinded to all clinical information other than the presence of lymphadenopathy. Lymph node short and long diameters, cortical thickness, and absence of fatty hilum were evaluated. Figure 1 shows a representative example of the assessment method. In the case of multiple lymph node involvement, the radiologist evaluated the lymph nodes that were biopsied.

### 2.5. Statistical Analysis

The association of clinical and imaging features with final diagnosis was assessed using the Chi-squared test for categorical variables and the Mann-Whitney U-test for continuous variables. Sensitivity, specificity, accuracy, positive predictive value (PPV), and negative predictive value (NPV) of clinical findings, US imaging findings, and US-guided sampling for distinguishing benign from malignant lymph nodes were calculated. For continuous variables, we performed receiver operating characteristic (ROC) curve analysis to calculate the area under the ROC curve (AUC) for diagnostic performance. Then, the optimal cutoff value closest to the upper left corner was derived.

All calculations were performed using the Statistical Package for the Social Sciences, version 24 (IBM, NY, USA). *p* values < 0.05 were considered statistically significant.

## 3. Results

Of the 3600 patients who were examined by US, 152 patients were diagnosed with lymphadenopathy and underwent US-guided FNA or CNB of an axillary lymph node. Of these 152 patients, 97 were excluded as they had newly diagnosed breast cancer (*n* = 90) or axillary lymph node recurrence of breast cancer within a year (*n* = 7). Finally, 55 patients (47 female (85.5%)) were included in this study.

Table 1 shows the characteristics of patients. Twenty-one patients (38.2%) had benign lesions and 34 patients (61.8%) had malignant lesions. None of the patients developed any complications after US-guided sampling, as assessed by physical examination.

The mean age (± standard deviation) of patients was 63.6 ± 14.4 years. Thirty-one patients (56.3%) had a history of cancer, 19 patients (34.5%) had a history of breast cancer, and 8 patients (14.5%) had a history of other diseases that can cause lymphadenopathy (rheumatoid arthritis or sarcoidosis). The most common indication for US-guided sampling was an abnormal lymph node detected by positron emission tomography (PET) (23, 41.2%), followed by an abnormal lymph node detected by computed tomography (CT) (13, 23.6%), awareness of axillary mass (10, 18.2%), and an abnormal lymph node detected by US (9, 16.4%).

On the US, the mean (±SD) long axis, short axis, long-short axis ratio, and cortical thickness of the lymph node were 19.1 ± 8.9, 11.4 ± 8.1, 2.50 ± 3.13, and 8.3 ± 6.2 mm, respectively. Twenty-two patients (40.0%) underwent FNA and 33 patients (60.0%) underwent CNB.

Older age (*p* = 0.034), history of non-cancerous disease that can cause lymphadenopathy (*p* = 0.002), palpable axillary mass (*p* = 0.003), larger short or long axis (*p* = 0.046, 0.007), thicker cortex (*p* < 0.001), and absence of hilum (*p* < 0.001) were significantly associated with malignancy.

Table 2 shows the results of US-guided FNA and CNB and the final diagnosis. On FNA, 11 patients had class I or class II lesions that were finally diagnosed as benign, while 9 patients had class V lesions that were finally diagnosed as malignant. Two patients had class III lesions; one of these patients had unchanged lymph nodes and was finally diagnosed as having a benign lesion, whereas the other had enlarged lymph nodes and was diagnosed as having a malignant lesion on follow-up.

On CNB, nine patients were found to have benign lesion and 23 patients were found to have malignant lesion; these were finally diagnosed as benign and malignant, respectively. One patient had to undergo additional excisional biopsy due to an inadequate specimen; this patient was diagnosed as having a malignant lesion (malignant lymphoma). Although most cases were diagnosed by hematoxylin-eosin staining alone, some cases were immunostained at the discretion of the pathologist.

Table 3 shows the diagnostic performance of physical examination, US imaging findings, and US-guided sampling. The diagnostic accuracy of palpable axillary mass was 0.691. The diagnostic accuracy of US imaging findings was highest for absence of hilum (0.800), followed by cortical thickness (0.782), short axis (0.762), and long axis (0.655). The diagnostic accuracy of FNA, CNB, and FNA + CNB was high (sensitivity, specificity, and accuracy of 0.909, 0.900, and 0.917 for FNA, 0.958, 1.000, and 0.970 for CNB, and 0.941, 0.952, and for FNA + CNB, respectively).

**Figure 2:**
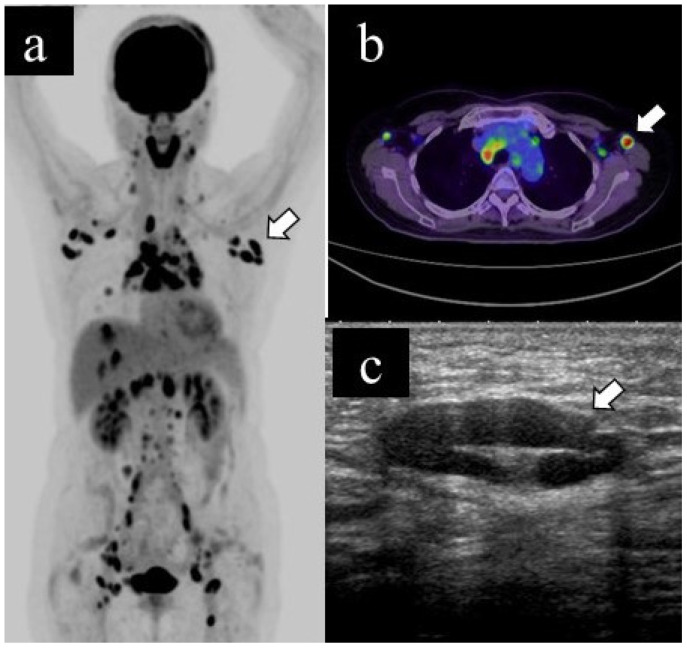
Representative case of benign lymphadenopathy. (**a**) Maximum-intensity projection (MIP), (**b**) transaxial 18F-FDG PET/CT image and (**c**) US image of benign lymphadenopathy.

**Figure 3:**
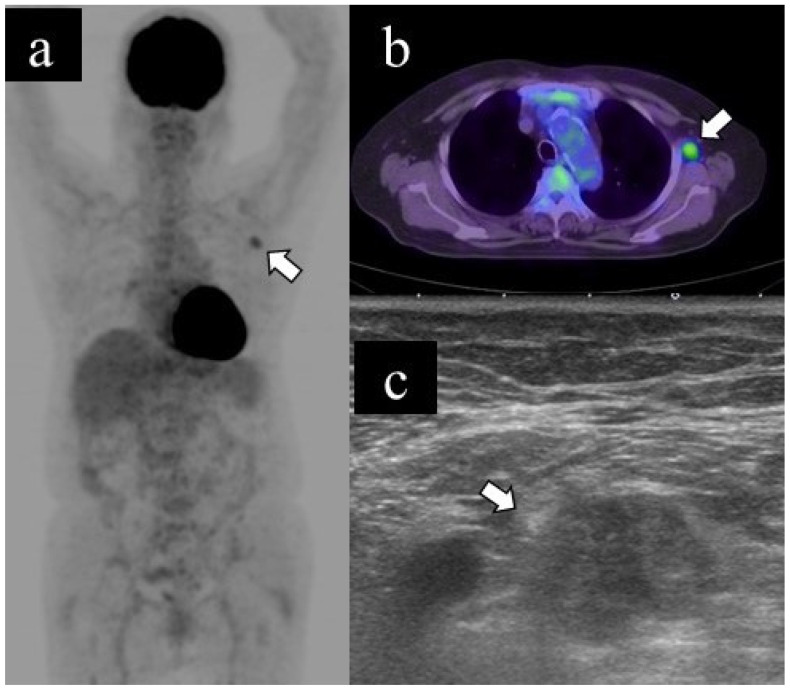
Representative case of malignant lymphadenopathy. (**a**) Maximum-intensity projection (MIP), (**b**) transaxial 18F-FDG PET/CT image and (**c**) US image of malignant lymphadenopathy.

Figure 2 and Figure 3 show representative cases of benign and malignant lymphadenopathy, respectively.

The patient, in her 60s, had cutaneous sarcoidosis and underwent a PET scan as a screening test. The results showed lymphadenopathy and abnormal uptake throughout the body, including the left axillary lymph node (a. b, arrow). US examination revealed left axillary lymphadenopathy (long diameter: 25.2 mm, short diameter: 8.9 mm, cortical thickness: 4.1 mm, and presence of fatty hilum) (b, arrow); subsequently, she underwent US-guided CNB. The pathological diagnosis was sarcoidosis. Spontaneous shrinkage of the lymph nodes was observed on follow-up.

The patient, in her 70s, underwent a PET scan for follow-up after renal carcinoma surgery, which revealed an unexpected abnormal uptake in the left axillary lymph node (a. b). US examination revealed lymphadenopathy (long diameter: 21.8 mm, short diameter: 13.1 mm, cortical thickness: 13.1 mm, loss of fatty hilum) (c); subsequently, she underwent US-guided FNA. The cytological classification was class V. The patient underwent axillary lymph node dissection and was finally diagnosed with axillary lymph node metastasis of renal carcinoma.

## 4. Discussion

In this study, patients with lymphadenopathy who underwent US-guided sampling were investigated. The clinical and imaging correlates of malignant lesions were examined and the diagnostic performance of US-guided FNA and CNB was assessed. Larger short and long axis diameters, thicker cortex, and the absence of hilum of the lymph nodes on the US showed a significant association with malignancy. In particular, thicker cortex and absence of hilum were strongly associated with malignancy (diagnostic accuracy for distinguishing between benign and malignant lesions: 0.782 and 0.800, respectively). Several studies have investigated the use of US for preoperative diagnosis of axillary lymph node metastasis in patients with breast cancer. Normal axillary lymph nodes are typically oval in shape, have a thin and uniform cortex, smooth margins, and a discernable central fatty hilum [4,5], whereas large lymph node diameter, focal or diffuse cortical thickening (>3 mm), abnormal morphology, and absence of fatty hilum are associated with malignancy [4,5]. According to a study, cortical and hilar changes are more important than lymph node size [12], which is consistent with our results. Although we did not use color or power Doppler in this study, the use of these technologies allowed us to obtain blood flow information, in addition to morphological information, and also allowed us to evaluate the absence of the hilum of the lymph nodes in detail, which may increase the performance of lymph node diagnosis. When making a final diagnosis with US, it is necessary to consider multiple parameters, rather than individual parameters. This study was not able to verify multiple parameters because of the small number of cases; however, in the next study, we would like to investigate whether the combination of multiple parameters contributes to the diagnostic performance.

Recent advances in computing have helped leverage the artificial intelligence (AI) technology for image classification; several studies have applied AI for evaluation of breast US imaging for diagnostic purposes [13,14,15,16]. Future prospects for use of AI for evaluation of US findings of axillary lymph nodes appear promising.

Clinically, palpable axillary mass and older age were associated with malignancy, whereas history of non-cancerous diseases that can cause lymphadenopathy (such as sarcoidosis and rheumatoid arthritis) was associated with benign disease. Male sex and history of malignancy tended to show a greater propensity for lymph node metastases, although the difference was not statistically significant. It is important to consider these clinical characteristics (in addition to imaging findings) for diagnosing axillary lymph nodes.

In this study, both FNA and CNB showed excellent diagnostic performance. The diagnostic accuracy of CNB (0.970) was slightly higher than that of FNA (0.917). In addition, no complications occurred during the study period, which is consistent with the minimally invasive nature of US-guided FAN and CNB.

In a study of 1353 patients with breast cancer, the sensitivity and specificity of CNB were 88% and 100%, respectively, while those of FNA were 74% and 100%, respectively [8]. Although the present study did not include patients with newly diagnosed breast cancer, we observed similar diagnostic performance of CNB and FNA in this study. According to a review of complications after US-guided FNA and CNB (*n* = 1272), 36 of 502 patients (7.1%) who underwent CNB and 13 of 975 patients (1.3%) who underwent FNA developed postoperative complications. In our cohort, the most common complication was pain, followed by hematoma and bruising [8].

US-guided FNA may be sufficient for differentiating between benign and malignant lesions in patients with lymphadenopathy; FNA is a less costly and less invasive examination than CNB. However, if the primary cancer is unknown, immunohistochemical staining may help detect the primary cancer lesion pathologically. Additionally, recently, tumor biomarkers have been used to guide treatment decision-making in several patients with breast and lung cancers [9,17]. In case the tumor biomarker is unknown, it is required to assess the characteristics of that tumor to determine an appropriate treatment strategy. Moreover, in case of decreasing efficacy of biomarker-based treatment, the status of the biomarker should be rechecked. In all these situations, it is preferable to obtain a larger specimen using CNB rather than FNA.

Similar to the present study, some previous studies have also examined axillary lymphadenopathy after excluding breast cancer patients. Schweb et al. [6] evaluated the pathological findings and the method of tissue harvesting in patients who had suspicious axillary lymph nodes but normal breast imaging findings. The authors reported that the majority of cases could be diagnosed using US-guided FNA and/or CNB. This study also showed similar results.

Garcia-Reyes et al. [10] assessed the outcomes of US-guided FNA in patients with suspected axillary nodes who had not been diagnosed with any cancer. Of the 78 lymph nodes sampled, only 2 (2.6%) were malignant. The discrepancy with our study with respect to the proportion of malignant lesions is likely attributable to the differences with respect to the study population (we included patients with a history of cancer other than breast cancer). The decision to perform lymph node sampling in patients with axillary lymphadenopathy should be based on the patient’s background as well as the imaging findings.

Although flow cytometry results were not considered in this study, flow cytometry can help diagnose malignant lymphoma. Flow cytometry requires a fresh specimen immersed in saline; hence, if we are performing an axillary lymph node biopsy for suspected malignant lymphoma, we need to consider whether we should obtain an additional fresh specimen.

Some limitations of the present study should be acknowledged. First, this was a retrospective, single center study with a relatively small sample size. Second, there are no established criteria as to whether lymph nodes should be sampled or whether FNA or CNB should be the method of choice; therefore, the decision-making was at the discretion of the radiologist performing the examination. Third, the study used two different US devices and was performed by multiple radiologists. Due to the different image quality of the devices and different skills of the radiologists, standardized images for analysis were not available, which may have affected the results of the study. However, since the two US systems were from the same vendor and the radiologists were well-trained specialists, their influence on the study should be minimal. In the future, prospective large-scale studies are required to confirm the usefulness of US-guided sampling in patients with axillary lymphadenopathy.

In conclusion, US is useful to evaluate lymph nodes in patients with axillary lymphadenopathy. US-guided FNA and CNB play an important role in the diagnosis and management of these patients.

## 5. Conclusions

US is useful to evaluate lymph nodes in patients with axillary lymphadenopathy. US-guided FNA and CNB play an important role in the diagnosis and management of these patients.

## Figures and Tables

**Table 1:** Characteristics of patients.

Number of Patients		All *n* = 55	Benign *n* = 21	Malignant *n* = 34	*p*
Clinical information					
Gender	Male/Female	8/47	1/20	7/27	0.112
Age	(y)	63.6 ± 14.4	59.2 ± 11.7	66.3 ± 15.3	0.034
History of cancer	Yes/No	31/24	9/12	22/12	0.112
History of breast cancer	Yes/No	19/36	6/15	13/21	0.464
History of other diseases *	Yes/No	8/47	7/14	1/33	0.002
Palpable axillary mass	Yes/No	27/28	5/16	22/12	0.003
Indication					
Awareness of axillary mass	(*n*)	10	3	7	
Abnormal LN on PET	(*n*)	23	8	15	
Abnormal LN on CT	(*n*)	13	4	9	
Abnormal LN on US	(*n*)	9	6	3	
US findings and US-guided sampling					
Long axis	(mm)	19.1 ± 8.9	16.8 ± 8.7	20.6 ± 8.8	0.046
Short axis	(mm)	11.4 ± 8.1	10.0 ± 10.0	12.2 ± 6.7	0.007
Long-short axis ratio		2.50 ± 3.13	2.46 ± 2.38	2.52 ± 3.55	0.943
Cortical thickness	(mm)	8.3 ± 6.2	5.8 ± 6.6	9.8 ± 5.5	<0.001
Absence of hilum	Yes/No	25/30	1/20	24/10	<0.001
Biopsy method	FNA/CNB	22/33	12/9	10/24	0.237

LN, Lymph node; CTD, Connective tissue diseases; US, Ultrasound; CT, Computed tomography; PET, Positron emission tomography. * Other diseases included sarcoidosis and rheumatoid arthritis, which can cause lymphadenopathy. Data are presented as mean ± standard deviation. *p* < 0.05 as statistically significant.

**Table 2:** Cytology and histology results and final diagnosis.

FNA	Final Diagnosis		CNB	Final Diagnosis	
Cytology	Benign (*n* = 12)	Malignant (*n* = 10)	Histology	Benign (*n* = 9)	Malignant (*n* = 24)
Class I	8	0	Benign	9	0
Class II	3	0			
Class III	1	1	Malignant	0	23
Class IV	0	0			
Class V	0	9			
Insufficient	0	0	Insufficient	0	1
	Normal 9 Reactive 3	Metastasis 10-Breast 6 -Renal 1-Uterine 1-Cervix 1-Esophagus 1		Reactive 4 Normal 2 Sarcoidosis 2 MTX-LPD 1	ML 8 Metastasis 16-Breast 7 -Lung 3-Ovary 2-Cervix 2-Tongue 1-Liver 1

FNA, Fine needle aspiration; CNB, Core needle biopsy; ML, Malignant lymphoma; MTX-LPD, Methotrexate-associated lymphoproliferative.

**Table 3:** Diagnostic performance of clinical information, ultrasound imaging findings, and ultrasound-guided sampling.

	Cutoff Value	Sensitivity	Specificity	Accuracy	PPV	NPV	AUC
Palpable axillary mass		0.647	0.762	0.691	0.815	0.571	
Long axis diameter	21.0 mm	0.500	0.905	0.655	0.895	0.528	0.661
Short axis diameter	7.0 mm	0.882	0.571	0.762	0.769	0.750	0.718
Cortical thickness	5.2 mm	0.824	0.714	0.782	0.824	0.714	0.803
Absence of hilum		0.706	0.952	0.800	0.960	0.667	
FNA		0.909	0.900	0.917	0.900	0.917	
CNB		0.958	1.000	0.970	1.000	0.900	
FNA + CNB		0.941	0.952	0.945	0.970	0.909	

FNA, Fine needle aspiration; CNB, Core needle biopsy; PPV, Positive predictive value; NPV, Negative predictive value; AUC, Area under the curve.

## Data Availability

All available data are presented within the article or are available on request from the corresponding author.
